# Nonlinear and Sex-Specific Associations of Vitamin D Metabolites with Inflammatory Blood Markers in 125,537 Adults †

**DOI:** 10.3390/nu17233670

**Published:** 2025-11-24

**Authors:** Xitong Li, Xin Chen, Yvonne Liu, Jingyun Wang, Carl-Friedrich Hocher, Christoph Reichetzeder, Saban Elitok, Bernhard K. Krämer, Anne Schönbunn, Cornelia Doebis, Katrin Huesker, Volker von Baehr, Berthold Hocher

**Affiliations:** 1 Fifth Department of Medicine (Nephrology/Endocrinology/Rheumatology/Pneumology), University Medical Center Mannheim, University of Heidelberg, 69120 Mannheim, Germany; lixitong0729@gmail.com (X.L.); xin.chen@charite.de (X.C.); yvonne.liu@charite.de (Y.L.); wjy6263@126.com (J.W.); hocherc@gmail.com (C.-F.H.); bernhard.kraemer@umm.de (B.K.K.); 2 Department of Nephrology, Charité Universitätsmedizin Berlin, 10117 Berlin, Germany; 3 Institute for Clinical Research and Systems Medicine, Health and Medical University, 14467 Potsdam, Germany; christoph.reichetzeder@uni-potsdam.de (C.R.); saban.elitok@klinikumevb.de (S.E.); 4 Department of Nephrology and Endocrinology, Klinikum Ernst von Bergmann, 14467 Potsdam, Germany; 5 Institute of Medical Diagnostics (IMD), 12247 Berlin, Germany; a.schoenbrunn@imd.berlin.de (A.S.); c.doebis@imd-berli.de (C.D.); k.huesker@imd-berlin.de (K.H.); v.vonbaehr@imd-berlin.de (V.v.B.); 6 Reproductive and Genetic Hospital of CITIC-Xiangya, Changsha 410008, China; 7 School of Medicine, Central South University, Changsha 410078, China

**Keywords:** 25-hydroxyvitamin D [25(OH)D], 1,25-dihydroxyvitamin D [1,25(OH)_2_D], inflammatory blood markers

## Abstract

Background: Vitamin D is increasingly recognized as a key immunomodulatory nutrient, influencing innate and adaptive immune responses. While 25-hydroxyvitamin D [25(OH)D] is widely used to assess vitamin D status, the active form, 1,25-dihydroxyvitamin D [1,25(OH)_2_D], may exert distinct effects on immune function. This study investigates the concentration-dependent and sex-specific relationships of both vitamin D metabolites with systemic inflammatory markers in a large clinical cohort. Objectives: To characterize the associations of 25(OH)D and 1,25(OH)_2_D with total white blood cell (WBC) count and leukocyte subtypes, including neutrophils, lymphocytes, monocytes, eosinophils, and basophils. Methods: We conducted a retrospective cross-sectional analysis of 125,537 adults (37.9% male; age 18–89 years) from routine laboratory diagnostics collected between 2014 and 2020 in Germany. Serum 25(OH)D and 1,25(OH)_2_D were measured using standardized chemiluminescent immunoassays. Inflammatory markers were assessed via automated hematology. Multivariable-adjusted linear and non-linear regression models were used to assess associations, adjusting for age, sex, and season. Results: **Neutrophils and Monocytes**: Displayed **U-shaped associations** with both 25(OH)D and 1,25(OH)_2_D. Neutrophil counts were **lowest at 25(OH)D levels of ~40–60 nmol/L** and increased significantly at both lower and higher extremes (*p* < 0.001). **Lymphocytes**: **Inverse relationship with 25(OH)D** (*p* < 0.001), and an **inverse U-shaped relationship with 1,25(OH)_2_D**, peaking at ~90 pmol/L, with counts decreasing at both lower and higher levels (*p* < 0.001). Sex-specific analysis revealed that the relationship between 1,25(OH)_2_D and lymphocyte count remained **independent only in men**, **Eosinophils and Basophils**: Demonstrated **consistently negative correlations** with both forms of vitamin D across all concentration ranges (*p* < 0.001). Conclusions: Our findings reveal distinct, concentration-dependent associations between vitamin D metabolites and leukocyte profiles, with evidence for nonlinear and sex-specific immunological effects. Both low and high levels of 25(OH)D and 1,25(OH)_2_D were linked to increased neutrophil and monocyte counts, suggesting that vitamin D excess, like deficiency, may be linked to low-grade inflammation. These data are hypothesis-generating and suggest that personalized monitoring of vitamin D status may be relevant for future research on immune health, particularly in populations at risk for inflammatory or metabolic disease, but they do not provide a basis for clinical decision-making.

## 1. Introduction

Vitamin D, long recognized for its role in calcium homeostasis and skeletal health [1], has emerged in recent decades as a key modulator of immune function. Vitamin D circulates in two major forms—25-hydroxyvitamin D [25(OH)D], the primary biomarker of vitamin D status, and 1,25-dihydroxyvitamin D [1,25(OH)_2_D], the biologically active metabolite. While 25(OH)D largely reflects sun exposure and dietary intake, circulating 1,25(OH)_2_D is tightly regulated by hormonal feedback involving PTH, FGF-23, and calcium. Therefore, unusually elevated levels of 1,25(OH)_2_D may indicate underlying metabolic or neoplastic conditions rather than increased vitamin D intake. Beyond its metabolic regulation, vitamin D functions both as a secosteroid hormone and a micronutrient, influencing a wide range of immunological pathways through its interaction with the vitamin D receptor (VDR) expressed in multiple immune cells, including macrophages, dendritic cells, and T lymphocytes [2]. Accumulating evidence suggests that vitamin D plays a central role in controlling inflammatory responses and maintaining immune tolerance. Deficiency or dysregulation of vitamin D metabolism has been associated with an increased risk of respiratory infections, autoimmune diseases, and chronic inflammatory conditions [3,4].

Among the most accessible biomarkers for systemic inflammation are C-reactive protein (CRP), white blood cell (WBC) count, as well as leukocyte subtypes such as neutrophils, lymphocytes, monocytes, eosinophils, and basophils. These cells each play specialized roles: neutrophils respond rapidly to infections; lymphocytes orchestrate adaptive immunity; monocytes serve as precursors for tissue macrophages and antigen-presenting cells; eosinophils mediate responses to allergens and parasites; and basophils contribute to hypersensitivity reactions [5,6]. As vitamin D interacts with multiple components of the immune system, examining its relationships with these leukocyte subtypes may offer deeper insights into its immunomodulatory potential.

Previous studies have generally reported an inverse correlation between serum vitamin D levels and CRP or WBC counts [7]. However, our own prior analyses suggest that this relationship may be nonlinear—specifically, U-shaped—with inflammatory markers initially decreasing and subsequently increasing at high vitamin D concentrations [8]. While the vitamin D-CRP relationship is relatively well established, far fewer studies have focused on the leukocyte subtypes. Notably, a study by Maghbooli et al. showed that vitamin D supplementation lowered the neutrophil-to-lymphocyte ratio (NLR), a marker of inflammation and disease severity [9]. Other research has suggested a significant inverse correlation between vitamin D and eosinophils, particularly in asthma models [10,11], whereas the relationship with lymphocytes appears inconsistent [12]. In monocytes, vitamin D has been shown to influence epigenetic programming, macrophage differentiation, and innate immune training [13,14]. Nevertheless, population-based data examining these dynamics remain scarce.

Furthermore, the current literature largely focuses on 25(OH)D, the inactive circulating precursor of vitamin D, while overlooking 1,25(OH)_2_D, its active hormonal form. This distinction, however, is critical, as the two metabolites may exert differing biological effects. Moreover, emerging evidence points to sex-specific differences in immune responses to vitamin D, potentially modulated by sex hormones, which warrants systematic investigation [15].

## 2. Materials and Methods

### 2.1. Study Population

The study was conducted in full compliance with the Declaration of Helsinki and received approval from the Institutional Review Board of the Potsdam Institute for Medical Diagnostics in Berlin (Approval Number: IMD-2023-09; Date of Approval: 4 October 2023). All data utilized in this research underwent internal quality assessment and were anonymized, thereby eliminating the need for patient consent. The confidentiality and privacy of patient information were rigorously safeguarded throughout the study.

The study, conducted between March 2014 and July 2020, analyzed data from 455,062 patients. Blood samples were collected from healthcare facilities across Germany and subsequently sent to the Institute for Medical Diagnostics (IMD) in Potsdam, Berlin, for clinical analysis. The inclusion criteria for the study were as follows: (1) precise measurement of 25(OH)D and/or 1,25(OH)_2_D concentrations, and (2) evaluation of at least one of the patients’ inflammatory response parameters (WBC, CRP, neutrophils, eosinophils, basophils, lymphocytes, monocytes) four weeks before or after the vitamin D measurements. The exclusion criteria included the following:

(1) Patients with elevated CRP levels (>5 mg/L).
(2) Pregnant women.
(3) We also excluded cases whose differential blood counts showed morphological features suggestive of a haematological malignancy (e.g., circulating blasts, significant dysplasia).

After applying these inclusion and exclusion criteria, a total of 125,537 cases were included in the final analysis.

### 2.2. Clinical and Laboratory Parameters

The concentrations of both forms of vitamin D were measured using an Abbott Architect i2000 electrochemiluminescence immunoanalyzer (Abbott Laboratories, Wiesbaden, Germany). At IMD Berlin, the analysis of white blood cell subtypes—including lymphocytes, granulocytes, and eosinophils—is typically performed using a Complete Blood Count (CBC) with an automated differential. This test quantifies the total number of white blood cells and provides a detailed breakdown of the different subtypes. The automated differential is conducted using advanced hematology analyzers, such as the Sysmex XN-Series (Sysmex Corporation, Kobe, Japen). These instruments employ fluorescence flow cytometry, where cells are treated with specific reagents that label intracellular nucleic acids. As the cells pass through a laser beam, they scatter light at various angles, and the emitted fluorescence is measured. This process allows for the differentiation and counting of various white blood cell populations based on their size, internal complexity, and nucleic acid content. The resulting data is plotted on scattergrams, enabling precise identification and enumeration of cell types, including immature granulocytes. CRP was quantified employing standardized protocols established by the Berlin Institute for Medical Diagnostics (https://www.imd-berlin.de/ accessed on 29 May 2024). All clinical and laboratory data were subjected to rigorous quality assurance and control measures to ensure accuracy and reliability. All laboratory assays, including vitamin D and leukocyte measurements, were performed under ISO-certified quality control procedures, with daily calibration and participation in external quality assessment schemes.

### 2.3. Statistical Analyses

Data analysis was conducted using SPSS version 23.0 (IBM, Armonk, NY, USA), with correlation plots generated using GraphPad Prism version 8 (GraphPad Software Inc., San Diego, CA, USA). The significance threshold was set at *p* < 0.05. All variables, except gender, are expressed as mean ± standard deviation (SD). Considering the strong sunlight dependence and seasonal variability of vitamin D levels, as demonstrated in our previous study, the data were divided into two groups based on seasonal differences in mean vitamin D concentrations: (1) months with low mean vitamin D concentrations (January to April and December) and (2) months with high mean vitamin D concentrations (May to November) [8].

To investigate the relationship between vitamin D levels and inflammatory response parameters, the dataset was further divided into ten groups based on the concentration of the two vitamin D forms. Three models—linear, exponential, and quadratic—were fitted to evaluate the relationship curves between vitamin D levels and inflammatory response markers. We ultimately applied quadratic regression models. Model selection was based on improvements in the adjusted R^2^, together with visual inspection of the fitted curves. Standardized residuals were examined to identify potential outliers or influential observations, and no cases with excessive influence on the model estimates were detected. In addition, residual plots were visually inspected to verify model assumptions and to exclude curvature driven by a small fraction of extreme values. Multiple regression analysis was performed using vitamin D concentration as the dependent variable and sex, seasonal grouping, WBC, CRP, and the five WBC subtypes as independent variables.

Where nonlinear relationships were identified between vitamin D levels and inflammatory response parameters, adjustments were applied to individual parameters to account for both linear and nonlinear associations [14]. Finally, the analysis was repeated separately for males and females in two age groups (<50 and ≥50 years) using the same methodology. To account for potential nonlinearity, we applied quadratic regression models and restricted cubic splines, comparing them with linear and exponential fits. For visualization, vitamin D levels were divided into deciles; however, the main inferences were drawn from continuous regression models.

## 3. Results

**Figure 1:**
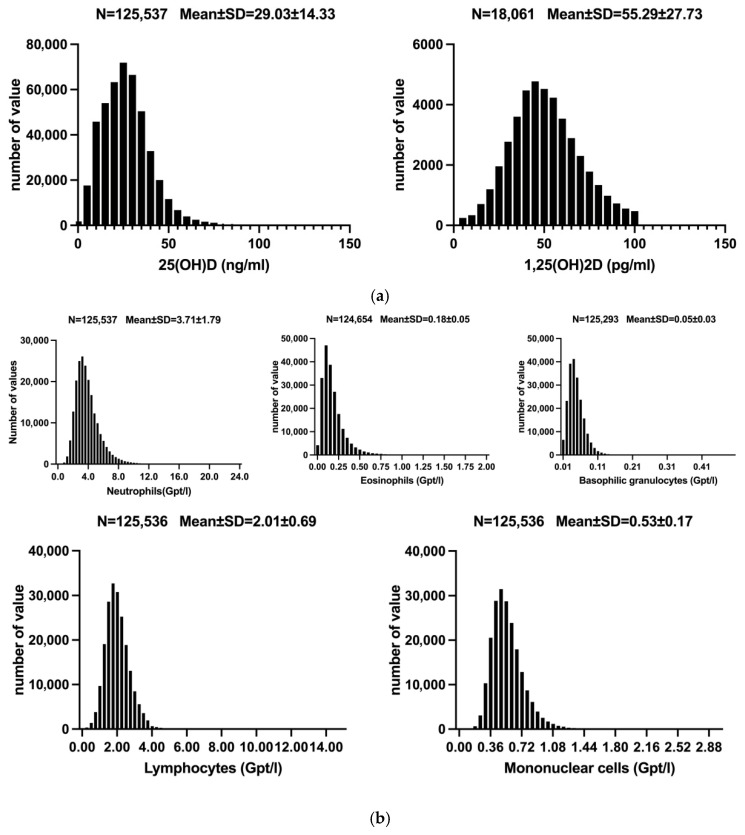
(**a**) **Distribution of Vitamin D.** Distribution of 25(OH)D and 1,25(OH)_2_D concentrations. Abbreviations: 25(OH)D: 25-hydroxy-vitamin D; 1,25(OH)_2_D: 1,25-dihydroxy-vitamin D; (**b**) **Distribution of different types of leucocytes.** Distribution of the five leukocyte subgroups.

A total of 125,537 cases were included in this study, of which 77,903 (62.1%) were female. The mean age of the participants was 55.38 ± 17.69 years. The mean concentrations of the two vitamin D forms were as follows: 25(OH)D at 29.03 ± 14.33 ng/mL and 1,25(OH)_2_D at 55.29 ± 27.73 pg/mL. The average concentrations of the primary inflammatory response markers were within their respective reference ranges, with a WBC concentration of 5.47 ± 2.03 Gpt/L and a CRP of 3.78 ± 1.29 mg/L. Detailed information on the five leukocyte subtypes and other parameters is presented in Table 1. The frequency distribution of the main study parameters is illustrated in Figure 1a,b.

To examine the relationship between vitamin D levels and inflammatory response parameters, three models—linear, exponential, and quadratic—were applied. Among these, the quadratic model demonstrated the highest R^2^ values for both 25(OH)D and 1,25(OH)_2_D with all five leucocyte subtypes, indicating a superior fit compared to the other two models. The results showed a statistically significant association, with *p* < 0.05, confirming that vitamin D levels are strongly correlated with these inflammatory response parameters (Table 2).

**Figure 2:**
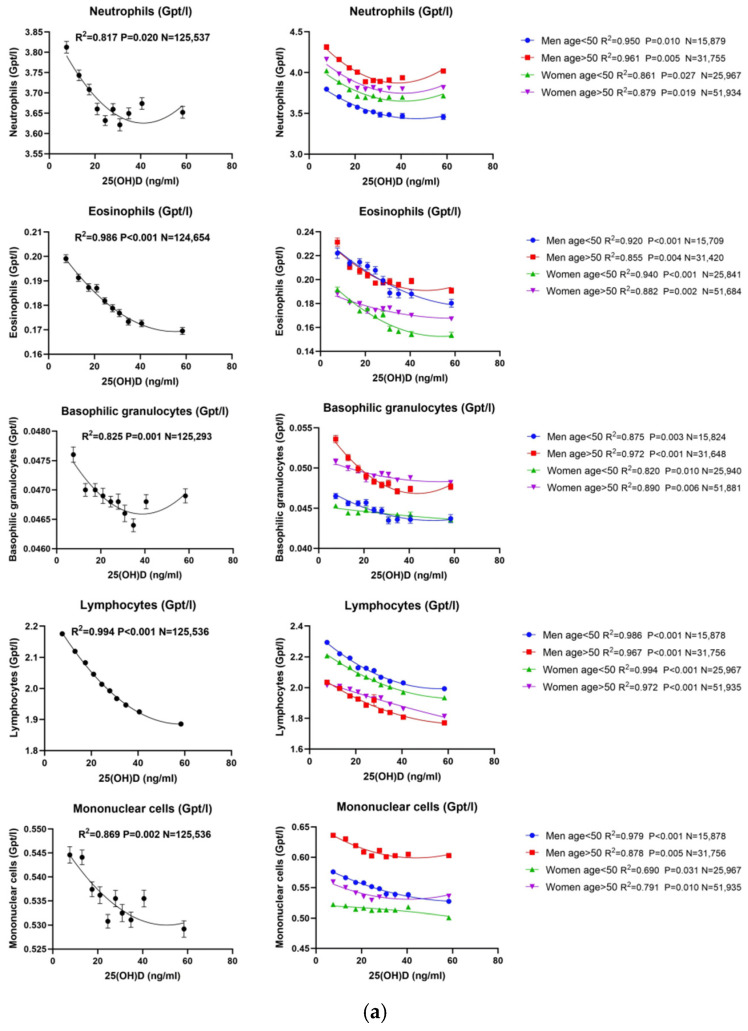

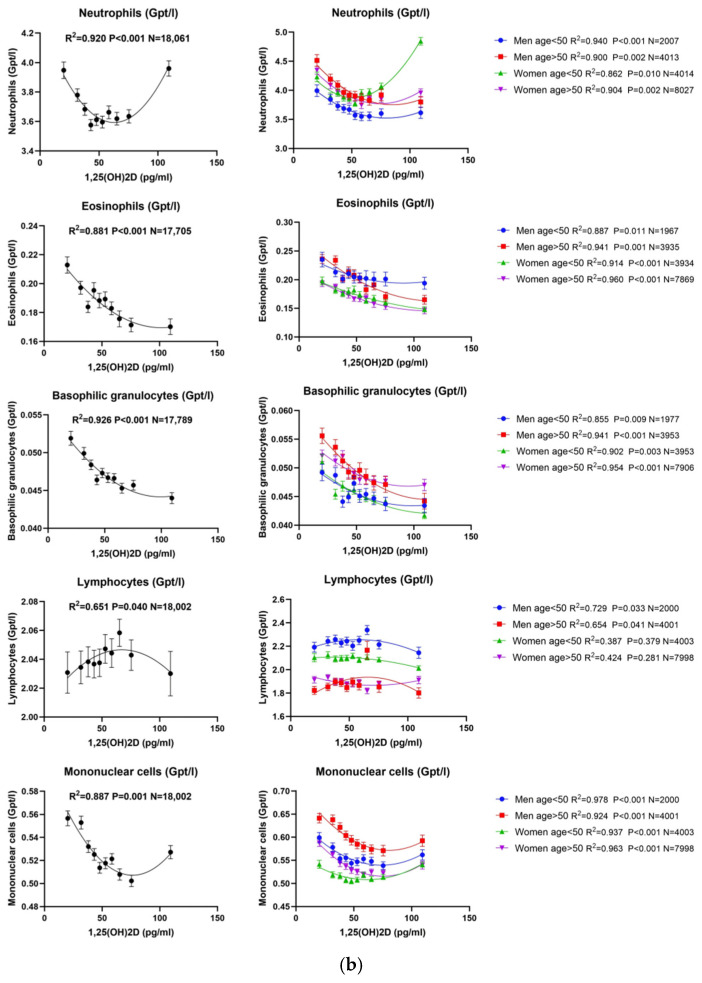
(**a**) **Correlation between 25(OH)D and different types of leucocytes**. Neutrophils and monocytes displayed U-shaped relationships, whereas lymphocytes, eosinophils, and basophils were inversely correlated. Data are presented as mean counts per decile of 25(OH)D, with quadratic regression lines fitted to the dataset. After gender and age grouping, the correlation between 25(OH)D and the five leukocyte classification cells was consistent with the overall data in both male and female groups, indicating that gender and age had no effect. Abbreviations: 25(OH)D: 25-hydroxy-vitamin D. (**b**) **Correlation between 1,25(OH)2D and different types of leucocytes**. Neutrophils and monocytes again exhibited a U-shaped association with 1,25(OH)_2_D levels, whereas eosinophils and basophils remained inversely correlated. Lymphocytes demonstrated an inverted U-shaped pattern, with counts increasing at moderate 1,25(OH)_2_D concentrations but declining at higher levels. Data are expressed as mean leukocyte counts per decile of 1,25(OH)_2_D, and quadratic regression models were fitted to the datasets. Multivariate regression analysis revealed that the association between lymphocyte counts and 1,25(OH)_2_D was significant and independent in males—particularly in younger men—but not in females across age groups. Abbreviations: 1,25(OH)_2_D: 1,25-dihydroxy-vitamin D.

We used three models (linear, exponential and quadratic) to fit the relationship curves between the two vitamin D forms and various inflammatory response parameters. Abbreviations: 25(OH)D: 25-hydroxy-vitamin D; 1,25(OH)_2_D: 1,25-dihydroxy-vitamin D; WBC: white blood cell; CRP: C-reactive protein. Subsequently, the dataset was divided into ten equal groups based on 25(OH)D and 1,25(OH)_2_D concentrations. To preliminarily screen for and exclude potential influences of metabolic or neoplastic disorders on circulating vitamin D metabolites, we conducted an exploratory analysis incorporating available biochemical markers. We found that 25(OH)D showed statistically significant associations with serum calcium and PTH; however, these relationships were weaker compared with those observed for 1,25(OH)_2_D. In contrast, the associations for 1,25(OH)_2_D were more pronounced and followed the expected endocrine physiological patterns—higher 1,25(OH)_2_D levels corresponded to slightly higher calcium and lower iPTH concentrations (Supplementary Figure S1). Taken together, these findings are consistent with normal vitamin D endocrine feedback and do not indicate overt disturbances in calcium–phosphate metabolism or neoplastic processes that might substantially confound the observed associations. Nevertheless, subclinical abnormalities cannot be entirely excluded. Correlation curves were then constructed to analyze the relationship between vitamin D levels and inflammatory response parameters. For 25(OH)D, the correlation curves with the five leukocyte subtypes showed that neutrophils and monocytes followed a similar U-shaped pattern, while eosinophils, basophils, and lymphocytes showed consistently negative associations (Figure 2a). Thus, both innate immune cells (neutrophils and monocytes) and adaptive/allergic-type cells (eosinophils, basophils, and lymphocytes) displayed distinct nonlinear or inverse relationships with vitamin D. For 1,25(OH)_2_D, the overall pattern resembled that of 25(OH)D, with some notable differences. Neutrophils and monocytes again displayed U-shaped associations, while eosinophils and basophils remained negatively correlated. In contrast, lymphocytes showed an inverse U-shaped relationship: counts increased with rising 1,25(OH)_2_D concentrations and declined at higher levels (Figure 2b). Taken together, these findings highlight that innate immune cells (neutrophils and monocytes) respond similarly to both vitamin D metabolites, whereas adaptive cells such as lymphocytes show a distinct and nonlinear pattern specifically with 1,25(OH)_2_D.”

To evaluate whether the correlations between inflammatory response parameters and vitamin D levels were independent, multiple regression analyses were conducted. Based on prior correlation analyses, both vitamin D levels and inflammatory response markers exhibited a quadratic relationship. To account for potential nonlinearity, the square root-transformed values of the two vitamin D metabolites were included as independent variables, while concentrations of various leukocyte subtypes were used as dependent variables. The results indicated that for 25(OH)D, all *p*-values associated with inflammatory parameters were below 0.05, suggesting that the associations between 25(OH)D and leukocyte subtypes were statistically independent. In contrast, for 1,25(OH)_2_D, the *p*-values for correlations with lymphocyte counts exceeded 0.05, implying that these associations may have been confounded by other factors (see Table 3). Table 3 summarizes the primary associations.

Finally, we performed a subgroup analysis based on gender and age. For 25(OH)D, all subgroups demonstrated correlations with all inflammatory parameters consistent with the overall trend (Figure 2a). Subsequent multiple regression analysis confirmed that the relationships between 25(OH)D and each inflammatory parameter were independent of other factors (Table 3). In contrast, the gender and age subgroup analysis for 1,25(OH)_2_D revealed some deviations from the overall trend. Specifically, in the male group, 1,25(OH)_2_D correlated with all inflammatory response parameters, following the same trend observed in the overall data. However, in the female group, 1,25(OH)_2_D lost its correlation with lymphocytes (*p* = 0.218), while maintaining correlations with the other inflammatory parameters consistent with the overall trend (Figure 2b). However, all correlations were also independent of age. Multiple regression analysis further showed that all inflammatory response parameters were independently correlated with 1,25(OH)_2_D in the male group. In the female group, however, similar to the findings from the overall dataset, lymphocytes did not exhibit independent correlations with 1,25(OH)_2_D (Table 3). As shown in Supplementary Table S1, the inverse association between 1,25(OH)_2_D and lymphocyte count was significant in males but not in females. The sex × 1,25(OH)_2_D interaction term was statistically significant (*p* < 0.001), confirming effect modification by sex.

## 4. Discussion

Our study, encompassing over 125,500 individuals, provides robust evidence that both 25(OH)D and 1,25(OH)_2_D are nonlinearly associated with key inflammatory blood markers. Unlike our previous work focusing on CRP and calcium–phosphate metabolism, the present analysis centers on leukocyte subtypes, thereby extending our understanding of vitamin D’s role from systemic protein markers to cellular immune profiles. We observed U-shaped relationships for neutrophils and monocytes, an inverse U-shaped pattern for lymphocytes (notably with 1,25(OH)_2_D), and consistent negative associations with eosinophils and basophils. Importantly, these associations were sex-specific, particularly in the case of lymphocyte counts. These findings underscore that both low and high vitamin D levels may be associated with low-grade inflammatory profiles, whereas moderate concentrations may be compatible with a more balanced immune phenotype. From a translational perspective, these observations are compatible with the notion that vitamin D may function not merely as a deficiency-sensitive micronutrient but as a bidirectional modulator of immune markers; however, this interpretation remains speculative. As such, these results may inform future studies on optimal vitamin D monitoring strategies, although specific clinical recommendations cannot be made based on observational data. These data are hypothesis-generating and may stimulate further research on how vitamin D status relates to inflammatory phenotypes in different clinical contexts, but they are not intended to inform therapeutic decision-making or management strategies.

### 4.1. Neutrophils and Monocytes

Our data revealed a distinct U-shaped relationship between both 25(OH)D and 1,25(OH)_2_D concentrations and the counts of neutrophils and monocytes, which closely mirrored the trend observed for total white blood cell counts. As crucial effector cells of the innate immune system, neutrophils and monocytes originate from myeloid progenitor cells in the bone marrow and serve as first responders in host defense through phagocytosis and cytokine release [15]. At moderate vitamin D levels (e.g., 25[OH]D ~40–60 nmol/L), neutrophil and monocyte counts were lowest, consistent with a potential immunoregulatory balance observed at moderate vitamin D levels. This may reflect vitamin D’s potential role in modulating cytokine secretion pathways, which has been suggested in experimental studies [16,17]. Additionally, 1,25(OH)_2_D promotes monocyte differentiation into macrophages or dendritic cells, enhancing their antigen-presenting and tissue-resident functions [18].

However, both deficient and excessive vitamin D levels were associated with increased neutrophil and monocyte counts. At low vitamin D levels, insufficient concentrations may be linked to heightened inflammatory signaling, while at high concentrations, potential pro-oxidative mechanisms have been reported, which could correspond to increased leukocyte counts observed here [19,20]. These observations highlight a potential biphasic association, suggesting that both insufficient and excessive vitamin D concentrations may coincide with altered immune profiles. These findings suggest that vitamin D status may be linked to innate immune tone and raise the hypothesis that neutrophil and monocyte counts could serve as markers of vitamin D-related immune variation. Prospective and interventional studies are needed to determine whether these observations have practical implications for supplementation monitoring.

### 4.2. Eosinophils and Basophils

Eosinophils and basophils are granulocytes derived from pluripotent stem cells in the bone marrow and are central players in allergic and inflammatory immune responses. Eosinophils are primarily implicated in chronic inflammatory processes, while basophils are key mediators of rapid, IgE-dependent allergic reactions [21]. Both cell types contribute to immune amplification by releasing potent mediators such as leukotrienes, histamine, and a range of cytokines, and by influencing the activity of T cells, mast cells, and monocytes.

In our study, both 25(OH)D and 1,25(OH)_2_D levels were consistently and inversely associated with eosinophil and basophil counts. This suggests that higher vitamin D levels are associated with lower eosinophil and basophil counts, consistent with an immunosuppressive role observed in prior studies. For eosinophils, vitamin D has been shown to inhibit tissue migration by downregulating chemokines such as eotaxin [22] and to suppress IL-5 production, a cytokine essential for eosinophil differentiation and survival [23]. Regarding basophils, previous research suggests that vitamin D may modulate IgE-mediated responses and basophil activation, which could help explain the negative associations observed in our data [24]. Furthermore, vitamin D is known to stabilize mast cells and basophils, reducing their release of histamine and other inflammatory mediators [25]. This inhibitory effect on degranulation contributes to attenuated recruitment and activation of eosinophils and basophils in allergic inflammation.

Together, these findings highlight the potential relevance of vitamin D status in allergic and eosinophilic disorders and suggest possible implications for conditions such as asthma, atopic dermatitis, and chronic rhinosinusitis; however, these implications remain speculative and require confirmation in dedicated clinical studies.

### 4.3. Lymphocytes

Lymphocytes exhibited the most complex response to vitamin D in our study. While 25(OH)D levels were negatively correlated with lymphocyte counts, 1,25(OH)_2_D displayed an inverted U-shaped trend: lymphocyte numbers initially increased with rising 1,25(OH)_2_D concentrations but declined beyond a certain threshold. As central players in adaptive immunity, lymphocytes encompass T cells, B cells, and natural killer (NK) cells, which collectively mediate immune memory, pathogen elimination, and tumor surveillance [26].

The inverse relationship between 25(OH)D and lymphocytes could be explained by previously reported proapoptotic effects at high concentrations, although this remains speculative, as well as suppression of Th1-mediated cytokines such as IL-2 and IFN-γ, which are key to proinflammatory T cell expansion [27,28]. In contrast, low to moderate concentrations of 1,25(OH)_2_D promote regulatory T cell (Treg) differentiation via FOXP3 gene induction, which supports both lymphocyte survival and an anti-inflammatory cytokine milieu (e.g., IL-10, TGF-β) [29,30]. In addition, 1,25(OH)_2_D enhances Th2-type responses by increasing IL-4, IL-5, and IL-13 production, fostering lymphocyte proliferation in early phases [31]. The upregulation of chemokines such as CCL19 and CCL21 at optimal 1,25(OH)_2_D concentrations further facilitates lymphocyte trafficking into peripheral blood [32].

However, at supraphysiological levels, 1,25(OH)_2_D shifts the balance toward excessive Treg proliferation, leading to suppression of other lymphocyte subsets and maintenance of immune quiescence [33]. Furthermore, both forms of vitamin D directly suppress B cell maturation and antibody production at high doses, contributing to a net decline in circulating lymphocytes [34]. These findings highlight that vitamin D status shows bidirectional associations with immune markers, which may be compatible with concentration-dependent regulation of immune cells; however, causal mechanisms cannot be inferred from the present data.

### 4.4. Gender and Age Analysis

Our study uncovered a complex interplay between sex, age, and the immunomodulatory effects of vitamin D, particularly concerning lymphocyte dynamics. The relationship between lymphocyte count and the active metabolite 1,25(OH)_2_D was not only sex-specific but also modulated by age. The most pronounced association was observed in younger men (<50 years). This pattern raises the possibility that the association between 1,25(OH)_2_D and lymphocyte counts may be most pronounced during the peak reproductive and immunologically active years in males, although this remains speculative. The heightened sensitivity in younger men may be explained by a synergistic hormonal milieu. During early and middle adulthood, men typically exhibit robust levels of androgens, which have been shown to have a relatively limited direct influence on FOXP3 gene activation in Tregs [35]. In this context, 1,25(OH)_2_D may serve as a primary endocrine signal for fine-tuning Treg differentiation and function. The steeper response in younger men suggests that their immune systems are more receptive to this form of regulation, potentially as a mechanism to balance strong adaptive immune responses. With advancing age, the decline in both androgen levels and possibly vitamin D receptor sensitivity may attenuate this relationship.

Conversely, the attenuated effect of 1,25(OH)_2_D on lymphocytes in women, consistent across age groups in the main analysis, points to a dominant role for estrogen in female immunoregulation. Estrogens can directly bind to estrogen response elements (EREs) in the FOXP3 promoter via estrogen receptors (ERα/ERβ), leading to transcriptional activation independent of vitamin D signaling [36]. This direct genomic pathway provides a potent, alternative mechanism for Treg regulation that may supersede or diminish the relative importance of 1,25(OH)_2_D-mediated pathways. The fluctuating levels of estrogen across the menstrual cycle in pre-menopausal women and their decline post-menopause create a dynamic regulatory landscape where the vitamin D signal is less discernible in population-level data.

Collectively, these observations suggest that the immunological correlates of vitamin D status may differ by sex and age, highlighting the importance of considering these variables in future hypothesis-driven studies. This highlights the critical importance of considering these biological variables to move towards a more personalized understanding of nutritional immunology. Future research should aim to integrate direct measurements of sex hormones with vitamin D metabolites and detailed immune phenotyping to fully elucidate these intricate interactions.

### 4.5. Clinical Implications

Our findings have several potential, but clearly hypothesis-generating, clinical implications. First, the observation that both 25(OH)D and 1,25(OH)_2_D are nonlinearly associated with leukocyte subtypes suggests that measurement of both metabolites—rather than 25(OH)D alone—may provide a more complete picture of vitamin D-related immune status. Second, the U-shaped associations with neutrophils and monocytes emphasize the need to avoid both deficiency and excessive supplementation, as either extreme may be associated with low-grade inflammation. The observed sex-specific association between 1,25(OH)_2_D and lymphocytes may suggest potential differences between men and women; however, these patterns are observational and could be influenced by unmeasured factors, including—but not limited to—sex hormones. Further studies are required to clarify these possibilities. These results support a more personalized approach to vitamin D supplementation, taking into account immune phenotype, sex, and clinical context. Such considerations may be particularly relevant in the management of allergic, autoimmune, and chronic inflammatory conditions, where vitamin D has established immunoregulatory roles. However, we must emphasize that the present findings are hypothesis-generating and suggest that vitamin D status may warrant individualized monitoring; nevertheless, these implications remain speculative and do not constitute actionable clinical recommendations.

### 4.6. Future Perspectives

The novel associations uncovered in this study, particularly the inverse U-shaped relationship of 1,25(OH)_2_D with lymphocytes and its sex-specific nature, open several compelling avenues for future research. To move from association to causation, interventional studies are required to determine if modifying vitamin D metabolite levels directly alters leukocyte profiles and immune function. Furthermore, our findings necessitate research that integrates data on sex hormones, vitamin D supplementation, and detailed medical histories to elucidate the underlying mechanisms and confirm the clinical relevance of these interactions in specific patient populations, such as those with autoimmune or allergic conditions.

### 4.7. Study Limitations

The present study, while more comprehensive, has several limitations. First, because of the cross-sectional design, causality cannot be inferred, and reverse causality—where inflammatory processes influence vitamin D metabolism—remains a plausible explanation. Second, despite adjustments for some confounders, several important and unmeasured variables—such as diet, physical activity, body mass index (BMI), smoking, comorbidities including solid tumors, medication use, vitamin D supplementation or intake, and socioeconomic status—may have substantially influenced both vitamin D status and leukocyte parameters, thereby introducing residual confounding that could have affected the observed associations. Furthermore, while the study identified a nonlinear association between vitamin D and inflammatory markers, the underlying mechanisms remain to be elucidated through experimental validation. Limitations were also evident in the sex-specific analysis; the lack of accurate sex hormone concentration data constrained a deeper investigation into the mechanisms underlying sex differences. Although exploratory analyses using calcium and PTH provided a preliminary screen for potential metabolic or neoplastic disorders affecting vitamin D metabolism, the database lacked definitive diagnostic information, and thus, such underlying conditions cannot be fully excluded. Although leukocyte subtypes were examined, the functional and phenotypic variations within these subtypes, such as distinct monocyte or lymphocyte populations, were not explored in detail. Moreover, we lacked data on vitamin D supplementation or intake, which may have influenced circulating levels and introduced additional residual confounding. Lastly, the study’s focus on a German population limits the generalizability of the findings to other populations with different genetic, environmental, or dietary backgrounds, and the lack of detailed information on participants’ place of residence may introduce residual confounding related to regional differences in sun exposure. Future research should aim to address these limitations by employing longitudinal or interventional designs, incorporating more comprehensive variable assessments, and conducting in-depth functional analyses to advance the understanding of the relationship between vitamin D and inflammation.

## 5. Conclusions

This study represents one of the largest population-based analyses to date examining the relationship between vitamin D metabolites and immune cell subtypes. Unlike previous studies, which often relied on small cohorts or focused narrowly on either 25(OH)D or inflammatory markers such as CRP, our investigation provides a comprehensive, bidirectional view of how both 25(OH)D and 1,25(OH)_2_D interact with differentiated leukocyte subsets, including neutrophils, monocytes, lymphocytes, eosinophils, and basophils.

A major strength of this study is its differentiation between the effects of 25(OH)D and the active form 1,25(OH)_2_D. We identified not only dose-dependent but also nonlinear (U-shaped and inverse U-shaped) associations, revealing that both insufficiency and excess of vitamin D may be linked to immune dysregulation. Furthermore, our gender-stratified and age-resolved analysis uncovered that the sex-specific effect of 1,25(OH)_2_D on lymphocyte dynamics is most pronounced in men under 50 years of age. This novel insight, confirmed in a low-inflammation subgroup, suggests a hormonal and life-stage modulation of vitamin D’s immunological action that is critically underrepresented in the current literature.

From a clinical and public health perspective, these findings support a more nuanced conceptual understanding of how vitamin D metabolites relate to immune cell profiles. Rather than directly informing thresholds or treatment strategies, our results are hypothesis-generating and suggest that future studies on vitamin D supplementation and monitoring should consider immune status, sex, and age. These insights may help to shape future research on autoimmune disease, infection susceptibility, allergy, and vaccine responsiveness, but they should not be interpreted as guidance for current therapeutic practice or public health recommendations.

## Figures and Tables

**Table 1:** Baseline characteristics of all study indicators.

	*N* (*n* = 125,537)	Mean ± SD	Reference Range
Sex	Males: 47,634 (37.9%) Females: 77,903 (62.1%)	-	-
Age (years)	125,537	55.38 ± 17.69	-
25(OH)D (ng/mL)	125,537	29.03 ± 14.33	30.00–100.00
1,25(OH)_2_D (pg/mL)	18,061	55.29 ± 27.73	19.90–79.30
WBC (Gpt/L)	125,537	5.47 ± 2.03	3.60–28.20
CRP (mg/L)	90,209	3.78 ± 1.29	<5.00
Neutrophils (Gpt/L)	125,537	3.71 ± 1.79	1.30–22.30
Eosinophils (Gpt/L)	124,654	0.18 ± 0.05	0.02–1.10
Basophilic granulocytes (Gpt/L)	125,293	0.05 ± 0.03	<0.35
Lymphocytes (Gpt/L)	125,536	2.01 ± 0.69	1.10–13.60
Mononuclear cells (Gpt/L)	125,536	0.53 ± 0.17	0.10–2.70

Abbreviations: 25(OH)D: 25-hydroxy vitamin D; 1,25(OH)_2_D: 1,25-dihydroxy vitamin D; WBC: white blood cell; CRP: C-reactive protein. Reference ranges are from the Berlin Institute for Medical Diagnostics (https://www.imd-berlin.de accessed on 29 May 2024).

**Table 2:** Curve estimation of serum vitamin D**25(OH)D**.

Parameters	Linear		Exponential		Quadratic	
	R^2^	*p*	R^2^	*p*	R^2^	*p*
Neutrophils (Gpt/L)	0.376	0.097	0.442	0.067	0.817	0.020
Eosinophils (Gpt/L)	0.867	<0.001	0.898	<0.001	0.986	<0.001
Basophilic granulocytes (Gpt/L)	0.649	0.017	0.723	0.008	0.825	0.001
Lymphocytes (Gpt/L)	0.899	0.001	0.921	<0.001	0.994	<0.001
Mononuclear cells (Gpt/L)	0.752	0.010	0.802	0.007	0.869	0.002
**1,25(OH)_2_D**						
Neutrophils (Gpt/L)	0.103	0.433	0.111	0.521	0.920	<0.001
Eosinophils (Gpt/L)	0.827	<0.001	0.844	<0.001	0.881	<0.001
Basophilic granulocytes (Gpt/L)	0.861	0.001	0.903	<0.001	0.926	<0.001
Lymphocytes (Gpt/L)	0.118	0.672	0.285	0.469	0.651	0.040
Mononuclear cells (Gpt/L)	0.398	0.098	0.472	0.068	0.887	0.001

**Table 3:** Multivariate regression for different types of leucocytes.

	ALL			MEN			WOMEN		
	*p*	B	95%CI	*p*	B	95%CI	*p*	B	95%CI
**Neutrophils**									
25(OH)D (ng/mL)	<0.001	−0.207	−0.309~−0.105	<0.001	−0.093	−0.106~−0.080	<0.001	−0.005	−0.010~−0.001
1,25(OH)2D (pg/mL)	<0.001	0.047	0.039~0.055	<0.001	−0.031	−0.052~−0.010	<0.001	0.007	0.004~0.010
**Eosinophils**									
25(OH)D (ng/mL)	0.041	−0.001	−0.003~0.000	0.027	−0.005	−0.010~0.000	0.047	−0.002	−0.004~−0.000
1,25(OH)2D (pg/mL)	<0.001	−0.010	−0.017~−0.003	<0.001	−0.003	−0.006~0.000	<0.001	−0.004	−0.009~−0.001
**Basophilic granulocytes**									
25(OH)D (ng/mL)	0.007	−0.010	−0.014~−0.006	<0.001	−0.001	−0.001~0.000	0.040	0.049	0.022~0.076
1,25(OH)2D (pg/mL)	<0.001	−0.004	−0.008~0.000	<0.001	−0.001	−0.002~−0.001	<0.001	−0.003	−0.004~−0.002
**Lymphocytes**									
25(OH)D (ng/mL)	<0.001	−0.047	−0.063~−0.031	<0.001	−0.042	−0.073~−0.011	<0.001	−0.001	−0.002~−0.001
1,25(OH)2D (pg/mL)	0.277	−0.013	−0.023~0.003	0.041	−0.045	−0.088~−0.002	0.059	−0.033	−0.066~0.000
**Mononuclear cells**									
25(OH)D (ng/mL)	<0.001	−0.004	−0.010~−0.002	<0.001	−0.003	−0.007~−0.001	<0.001	−0.001	−0.001~0.000
1,25(OH)2D (pg/mL)	0.005	−0.002	−0.005~−0.001	0.003	−0.007	−0.008~−0.006	0.002	−0.001	−0.002~0.000

Multivariable-adjusted associations of serum 25(OH)D and 1,25(OH)_2_D with differential WBC counts. Linear and non-linear regression models were fitted using restricted cubic splines. All models were adjusted for age, sex, and season as core confounders. Additional covariates (e.g., estimated glomerular filtration rate, calcium, CRP, and others) were tested in sensitivity analyses (see Supplementary Table S1 for full model specifications and results). Beta coefficients represent the direction and magnitude of the association per unit change in each vitamin D metabolite. Units: 25(OH)D (nmol/L); 1,25(OH)_2_D (pmol/L); Abbreviations: 25(OH)D: 25-hydroxy-vitamin D; 1,25(OH)_2_D: 1,25-dihydroxy-vitamin D; CRP: C-reactive protein; B: unstandardized coefficient B; 95%CI: confidence interval.

## Data Availability

The original contributions presented in this study are included in the article/Supplementary Material. Further inquiries can be directed to the corresponding author.

## Supplementary Materials

The following supporting information can be downloaded at: https://www.mdpi.com/article/10.3390/nu17233670/s1. Table S1: Sex-specific association of 1,25(OH)_2_D with lymphocyte counts and interaction term; Figure S1: Associations of vitamin D with serum calcium and Ipth.
