# Supplementary material for: Nonlinear and Sex-Specific Associations of Vitamin D Metabolites with Inflammatory Blood Markers in 125,537 Adults
Source: Nutrients. 2025 Nov 24;17(23):3670. doi: 10.3390/nu17233670 (PMC12694123; doi:10.3390/nu17233670)
Supplement: Supplementary file 1 [file nutrients-17-03670-s001.zip › nutrients-3998200-supplementary.pdf]

Supplementary Table S1. Sex-specific association of 1,25(OH)₂D with lymphocyte counts and interaction term

	P	B	95%CI
**Constant**	<0.001	0.227	0.216~0,238
**Gender**	<0.001	0.066	0.039~0.093
**Age(years)**	<0.001	–0.002	-0.002~-0.001
**Seasonal group**	0.018	0.012	0.002~0.022
**1,25(OH)$_2$D**	<0.001	-0.010	-0.016~-0.004
**1,25(OH)$_2$D × Sex**	<0.001	-0.018	-0.026~-0.010

Multivariate regression analysis showing the sex-specific association between 1,25(OH)$_2$D and lymphocyte count (dependent variable), with the inclusion of the sex interaction term.
The dependent variable was lymphocyte count. The model was adjusted for age and season of blood draw. Sex was coded as 0 = female and 1 = male. In this model, the coefficient of 1,25(OH)$_2$D indicates the slope in females, and the coefficient of the interaction term (1,25(OH)$_2$D × Sex) represents the difference in slopes between males and females. Therefore, the slope for males is the sum of B(1,25(OH)$_2$D) and B(interaction). A significant negative interaction ($p < 0.001$) indicates that the inverse association between 1,25(OH)$_2$D and lymphocyte counts is stronger in males.

Abbreviations:1,25(OH)$_2$D: 1,25-dihydroxy-vitamin D; B: unstandardized coefficient B; 95%CI: confidence interval.

Supplementary Figure S1. Associations of vitamin D with serum calcium and iPTH

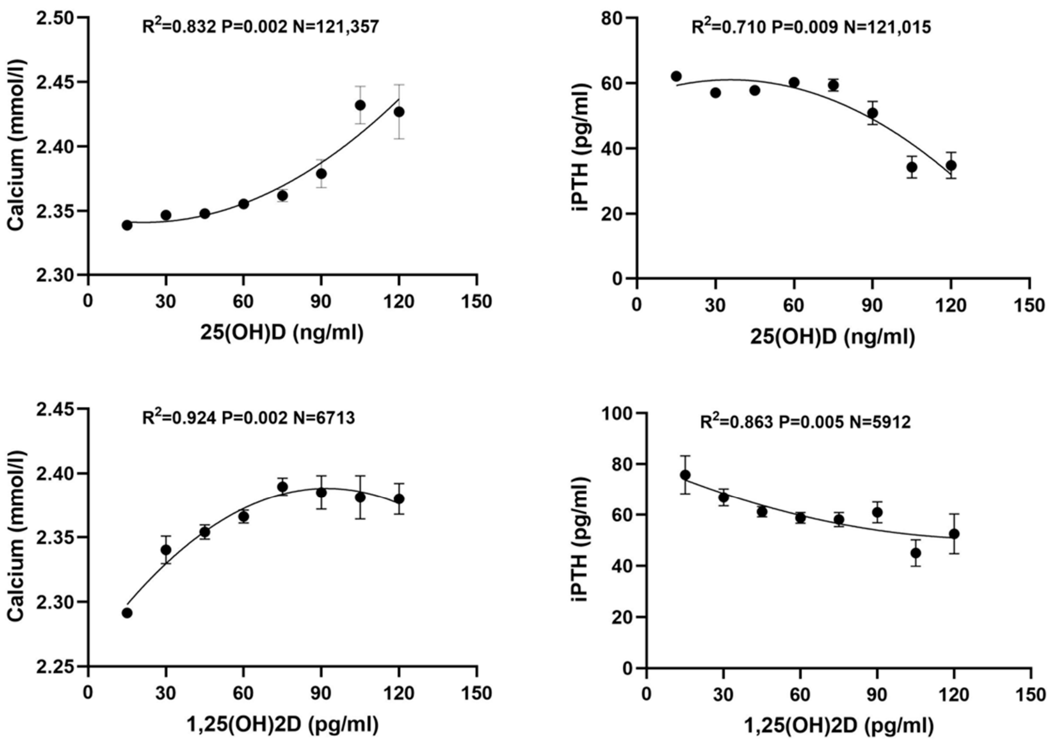

Compared with 25(OH)D, the associations of 1,25(OH)$_2$D with calcium and PTH were more pronounced, showing a physiological pattern in which calcium increased slightly and PTH declined with higher 1,25(OH)$_2$D levels.

These trends are consistent with the normal vitamin D endocrine feedback system and do not suggest major disturbances indicative of underlying metabolic or neoplastic disease

Abbreviations: 25(OH)D: 25-hydroxy vitamin D; 1,25$(OH)_2$D: 1,25-dihydroxy vitamin D;iPTH: intact Parathyroid Hormone.
